# WHO releases first-ever list of health-threatening fungi

**Published:** 2022-11

**25 October 2022** - WHO today published a report highlighting the first-ever list of fungal “priority pathogens” – a catalogue of the 19 fungi that represent the greatest threat to public health. The WHO fungal priority pathogens list (FPPL) is the first global effort to systematically prioritize fungal pathogens, considering the unmet research and development (R&D) needs and the perceived public health importance. The WHO FPPL aims to focus and drive further research and policy interventions to strengthen the global response to fungal infections and antifungal resistance.

Fungal pathogens are a major threat to public health as they are becoming increasingly common and resistant to treatment with only four classes of antifungal medicines currently available, and few candidates in the clinical pipeline. Most fungal pathogens lack rapid and sensitive diagnostics and those that exist are not widely available or affordable globally.

The invasive forms of these fungal infections often affect severely ill patients and those with significant underlying immune system related conditions. Populations at greatest risk of invasive fungal infections include those with cancer, HIV/AIDS, organ transplants, chronic respiratory disease, and post-primary tuberculosis infection.

Emerging evidence indicates that the incidence and geographic range of fungal diseases are both expanding worldwide due to global warming and the increase of international travel and trade. During the COVID-19 pandemic, the reported incidence of invasive fungal infections increased significantly among hospitalized patients. As the fungi that cause common infections (such as candida oral and vaginal thrush) become increasingly resistant to treatment, risks for the development of more invasive forms of infections in the general population are also growing.

“Emerging from the shadows of the bacterial antimicrobial resistance pandemic, fungal infections are growing, and are ever more resistant to treatments, becoming a public health concern worldwide” said Dr Hanan Balkhy, WHO Assistant Director-General, Antimicrobial Resistance (AMR).

Despite the growing concern, fungal infections receive very little attention and resources, leading to a scarcity of quality data on fungal disease distribution and antifungal resistance patterns. As a result, the exact burden of fungal diseases and antifungal resistance, are unknown, and the response is therefore undermined.

**Three priority categories**

The WHO FPPL list is divided into three categories: critical, high and medium priority. The fungal pathogens of in each priority category are so ranked primarily due to their public health impact and/or emerging antifungal resistance risk. While recognizing these critical pathogens as of public health concern globally, WHO emphases that the FPPL must be interpreted and contextualized carefully, as some endemic pathogens could be of more concern in their respective regional or local contexts.

**Need for more evidence and priority areas for action**

The authors of the report stress the need for more evidence to inform the response to this growing threat and to better understand the burden – both of disease and antifungal resistance. The report also highlights the urgent need for coordinated action to address the impact of antifungal use on resistance across the One Health spectrum and calls for expanding equitable access to quality diagnostics and treatments.

“We need more data and evidence on fungal infections and antifungal resistance to inform and improve response to these priority fungal pathogens” said Dr Haileyesus Getahun, WHO Director, AMR Global Coordination Department.

The FPPL report underscores strategies for policymakers, public health professionals and other stakeholders. The strategies proposed in the report are collectively aimed at generating evidence and improving response to these fungal priority pathogens including preventing the development of antifungal drug resistance. The primary recommended actions are focused on: (1) strengthening laboratory capacity and surveillance; (2) sustaining investments in research, development, and innovation; and (3) enhancing public health interventions for prevention and control.

“Countries are encouraged to follow a stepwise approach, starting with strengthening their fungal disease laboratory and surveillance capacities, and ensuring equitable access to existing quality therapeutics and diagnostics, globally” added Dr Haileyesus Getahun.

Resistance to antifungal medicines is partly driven by inappropriate antifungal use across the One Health spectrum. For example, injudicious use of antifungals in agriculture was linked to the rising rates of azole-resistant Aspergillus fumigatus infections. The report also calls for fostering WHO’s collaborative effort with the Quadripartite organizations and other partners, to address the impact of antifungal use on resistance across the One Health spectrum.

Available from: https://www.who.int/news/item/25-10-2022-who-releases-first-ever-list-of-health-threatening-fungi

